# Decoding Multi-Omics Signatures in Lower-Grade Glioma Using Protein–Protein Interaction-Informed Graph Attention Networks and Ensemble Learning

**DOI:** 10.3390/diagnostics15222894

**Published:** 2025-11-14

**Authors:** Murtada K. Elbashir, Afrah Alanazi, Mahmood A. Mahmood

**Affiliations:** Department of Information Systems, College of Computer and Information Sciences, Jouf University, Sakaka 72388, Saudi Arabia; mkelfaki@ju.edu.sa (M.K.E.); mamahmood@ju.edu.sa (M.A.M.)

**Keywords:** lower-grade glioma, multi-omics integration, graph attention network, protein–protein interaction network, biomarker discovery

## Abstract

**Background/Objectives**: Lower-grade gliomas (LGGs) are a biologically and clinically heterogeneous group of brain tumors, for which molecular stratification plays essential role in diagnosis, prognosis, and therapeutic decision-making. Conventional unimodal classifiers do not necessarily describe cross-layer regulatory dynamics which entail the heterogeneity of glioma. **Methods**: This paper presents a protein–protein interaction (PPI)-informed hybrid model that combines multi-omics profiles, including RNA expression, DNA methylation, and microRNA expression, with a Graph Attention Network (GAT), Random Forest (RF), and logistic stacking ensemble learning. The proposed model utilizes ElasticNet-based feature selection to obtain the most informative biomarkers across omics layers, and the GAT module learns the biologically significant topological representations in the PPI network. The Synthetic Minority Over-Sampling Technique (SMOTE) was used to mitigate the class imbalance, and the model performance was assessed using a repeated five-fold stratified cross-validation approach using the following performance metrics: accuracy, precision, recall, F1-score, ROC-AUC, and AUPRC. **Results**: The findings illustrate that a combination of multi-omics data increases subtype classification rates (up to 0.984 ± 0.012) more than single-omics methods, and DNA methylation proves to be the most discriminative modality. In addition, analysis of interpretability using attention revealed the major subtype-specific biomarkers, including UBA2, LRRC41, ANKRD53, and WDR77, that show great biological relevance and could be used as diagnostic and therapeutic tools. **Conclusions**: The proposed multi-omics based on a biological and explainable framework provides a solid computational approach to molecular stratification and biomarker identification in lower-grade glioma, bridging between predictive power, biological clarification, and clinical benefits.

## 1. Introduction

Diffuse glial brain tumors, also known as lower-grade gliomas (LGGs), are highly heterogeneous on the molecular, epigenetic, and clinical levels [1]. They are tumors of the central nervous system and are infiltrative, that is, represent about 15–20% of all primary brain tumors, and they occur more frequently in younger adults [2]. Among the most significant molecular stratifications in LGGs are mutations in the isocitrate dehydrogenase (IDH) genes and the presence or absence of 1p/19q co-deletion, which divides LGGs into subcategories, including IDHmut-codel, IDHmut-non-codel, and IDH wildtype (IDHwt) [3,4]. These subtypes vary enormously in terms of prognosis, therapeutic response, and biological behavior, and proper classification of the subtype is very important in diagnosis and treatment planning on an individual basis [5]. In addition to IDH and 1p/19q, additional genetic and epigenetic alterations (e.g., TP53 mutations, ATRX loss, DNA methylation patterns, and microRNA dysregulation) play a role in glioma pathogenesis and progression [5,6]. Multi-omics profiling has shown that these layers of molecular data can be used to gain complementary information on tumor biology, and that integrative computational methods are required to fully decode LGG heterogeneity [7]. Nevertheless, even though the correct LGG stratification is a clinically significant issue, the currently available diagnostic pipelines are frequently based on single-omics data, which may lead to misclassification and reduce their predictive power.

Traditional methods of subtype classification of LGGs are often based on single-omics data such as RNA expression, DNA methylation, or histopathology [8]. Although these methods have been effective in giving an insight into tumor biology, they tend to give one stream of information and thus fail to reflect the complex molecular interactions underlying gliomagenesis. As an example, gene expression profiles can reflect transcriptional programs, but do not reflect upstream regulatory responses like epigenetic and microRNA-based post-transcriptional regulation. Similarly, MGMT promoter methylation/methylation-based classifiers have been found to be helpful in prognosis, but alone they cannot fully address clinically relevant LGG subtypes [9]. Due to the emergence of high-throughput sequencing and profiling technologies, multi-omics datasets are becoming increasingly comprehensive. These datasets include transcriptomics, DNA methylation, microRNA expression, and sometimes proteomics that offer complementary insights into tumor biology [10]. A combined study of these modalities can be used to identify cross-layer regulatory processes, such as the silence of important tumor suppressors by DNA methylation, microRNA-mediated regulation of oncogenes, and how the integration of these processes can form transcriptomic phenotypes [11].

Recent research has shown that integrating multi-omics data can enhance the performance of classification significantly compared to single-omics approaches [12]. Multi-omics integration leverages the complementary strengths of different data types, since, unlike single modal models, multi-omics models can reduce the problems of data sparsity and noise, or incomplete biological coverage. To illustrate this, transcriptomic data may be involved in the identification of downstream gene activity, DNA methylation may be involved in the identification of epigenetic regulation, and microRNAs may be involved in the monitoring of post-transcriptional regulation. Coupled together, these modalities can overcome the ambiguities that occur when using one layer of information [13]. Not only do multi-omics methods provide greater predictive power but also biomarkers with an improved capacity to be replicated across independent cohorts [14]. Integrative signatures are also less prone to technical variability, batch effects, or platform-related biases which allow them to better generalize during cross-cohort validation. This strength is vital in LGGs, where the number of patients is relatively small and class imbalance (e.g., underrepresentation of IDH wildtype cases) is also another issue.

In addition to improving the prediction, multi-omics approaches also boost biological readability by connecting the changes in regulatory levels [15]. Indicatively, overexpressed oncogenes can be attributed to hypomethylation of their promoter, and silencing of their inhibitory microRNAs, which offers a mechanistic perspective unapprehend on unimodality analysis [16]. Notably, protein–protein interaction (PPI) networks provide an extra systems layer of view, in which molecular alterations are placed into context in functional pathway interaction modules [17,18]. This network-aware integration can identify central hub genes and signaling pathways that support gliomagenesis and can provide both new insights not limited to single features of molecules [19,20]. Such results support the significance of multi-omics integrations enhanced with PPI priors in forming the basis of precision oncology in LGGs to enhance the accuracy of the methodology and to discover the system’s tumor biology complexity.

In this work we introduce a protein–protein interaction (PPI)-aware Graph Attention Network (GAT) framework to decode multi-omics signatures in LGG. In contrast to the past methods that do not consider the structure of the biological network, our model combines RNA expression, DNA methylation, and microRNA expression into a biological-informed PPI graph so that the resulting network topology can capture known molecular relationships between genes. Although some recent works have modeled multi-omics data with graph neural networks (GNNs) or graph convolutional models, most of these models either (i) model each omics layer separately without utilizing biologically verified interaction priors, or (ii) build correlation-based graphs, which do not reflect functional relationship between genes. Our PPI-aware GAT framework has three major differences. First, it directly incorporates experimentally validated protein–protein interaction topology (STRING-based) into graph building, so that the flow of messages does not take place in arbitrary statistical associations but in biologically significant ones. Second, by creating node features based on constellations of integrated mRNA, miRNA, and DNA-methylation profiles, the model represents cross-layer regulatory dependencies among every gene. Third, the hybrid system with Random Forest (RF) feature importance and logistic stacking has better interpretability and stability than end-to-end GAT classifiers.

The pipeline used in the experiment consists of ElasticNet-based feature selection to select highly informative features among omics layers and SMOTE resampling to address the issue of class imbalance. Our meta-classifier is a ridge-regularized logistic regression that combines the strengths of the RF and GAT, and, thus, it uses the probabilistic output of both models and attempts to boost their robustness in classification. The performance is measured using repeated stratified cross-validation (five-fold cross-validation) and we employed the performance measures of accuracy, precision, recall, F1 score, ROC-AUC, and AUPRC. Moreover, the interpretability analysis that integrates attention scores from GAT and features importance from RF was applied to determine key genes that drive subtype discrimination. The analysis indicates new gene candidates with possible diagnostic and therapeutic significance. This transpiring biologically based and explainable multi-omics combination framework spans predictive performance and biological interpretability, providing a solid computational strategy for molecular stratification of LGG subtypes.

## 2. Materials and Methods

### 2.1. Datasets

Multi-omics datasets of lower-grade glioma (LGGs) were obtained using the GDC query function [21] in the GDC portal. The query was set to cover gene expression measurements, miRNA expression measurements, and DNA methylation data of the TCGA-LGG project, including both primary tumor and solid normal tissue samples. This allowed comparison of molecular and epigenetic changes in tumor and non-tumor tissues, revealing information on the changes regulating glioma. The GDCprepare function of the TCGAbiolinks R package (v2.28.4), which standardizes TCGA data to enable downstream analysis, was subsequently used to process the downloaded data. This will help in having correct data processing as the raw files will be modified into analysis-ready formats, as well as carry out critical preprocessing tasks such as normalization, filtering, and annotation to enhance the consistency. Integrated analyses across regulatory layers can be performed with the inclusion of several molecular profiles (gene expression, miRNA expression, and methylation).

To investigate transcriptional changes, the DESeq2 (v1.40.2) [22] was used, which involves the use of a generalized linear model to compare tumor and normal samples. The RowSums filter was used to filter the very low abundance or variability; only genes with a total count greater than 2 and present in more than four samples were retained. In the case of miRNA expression and DNA methylation, the limma package (v3.56.2) [23] was used, which involves the construction of linear models to determine the relationship between molecular features and experimental groups. Non-informative features are filtered out, and significantly changed features are identified by this approach.

Each omics layer was preprocessed and statistically modeled in a separate manner to capture their underlying data distribution. In case of the mRNA expression data represented by RNA-Seq, we utilized DESeq2, which estimates the count-based data based on a negative binomial distribution and includes variance stabilization and normalization of the size factor, which makes sure that with the help of this method, the dispersion is properly estimated in the samples. On the contrary, the miRNA and DNA methylation data were continuous and approximately normally distributed after log transformation and background-correction. Hence, we used the limma package, which uses linear modeling with empirical bayes variance moderation, a powerful method of microarray and methylation array data analysis with moderate sample sizes. Such a customized mix guarantees statistical consistency and maximum performance of any omics modality.

In lower-grade gliomas (LGGs), stratification of the samples was carried out into three molecularly characterized subtypes according to the WHO classification and TCGA marker studies of 2016: IDH-mutant with 1p/19q co-deletion (IDHmut-codel), IDH-mutant without co-deletion (IDHmut-non-codel), and IDH-wildtype (IDHwt). These subtypes represent the main molecular heterogeneity of LGGs and are closely related to the variance in prognosis, response to treatment, and biology. Particularly, IDHmut-codel tumors tend to have positive prognoses, and are associated with oligodendrogliomas; IDHmut-non-codel tumors are generally astrocytomas with intermediate prognoses. Conversely, the clinical and molecular characteristics of the IDH-wildtype subtype are associated with glioblastomas and are also associated with lower prognoses. This three-class model is extensively used in TCGA studies and has formed the basis of integrative multi-omics research of glioma pathophysiology and patient outcomes [3,24,25].

### 2.2. Data Annotation

All the three omics datasets were annotated in a systematic way to provide a uniform and biological interpretable annotation at the gene level across data modalities. In the case of gene expression data, the Ensembl gene identifiers were converted to official gene symbols and Entrez Gene IDs with the Ensembl BioMart service. Searches were made in batches to obtain the relevant annotations, and the expression matrix headers were revised to make sure that they were compatible with standardized nomenclature. In the case of miRNA data, annotations of expression profiles were performed by associating each miRNA to its main target gene based on experimentally validated miRNA-target interaction data within the miRTarBase (hsa_MTI) database. Identifiers were made unique via case normalization and removing suffixes; then, the most frequently reported target gene per miRNA was assigned. This allowed the ready incorporation of miRNA profiles into gene-level regulatory data. For DNA methylation data, the annotation of CpG sites was performed with the help of the reference Illumina HumanMethylation450k (hg19 build). Matcher CpG probes found in the dataset were aligned to annotation resource and their associated contexts (UCSC RefGene Name) were added. Where CpGs overlapped across several genes, redundancies were merged into unique sets of genes. The annotated datasets were further exported to undergo downstream analysis. Together, this annotation pipeline organized the RNA-Seq and miRNA and DNA methylation data into a shared gene-centric model, allowing the multi-omics data to be combined and analyzed by modeling them as a network.

### 2.3. The Hybrid Framework

**Figure 1:**
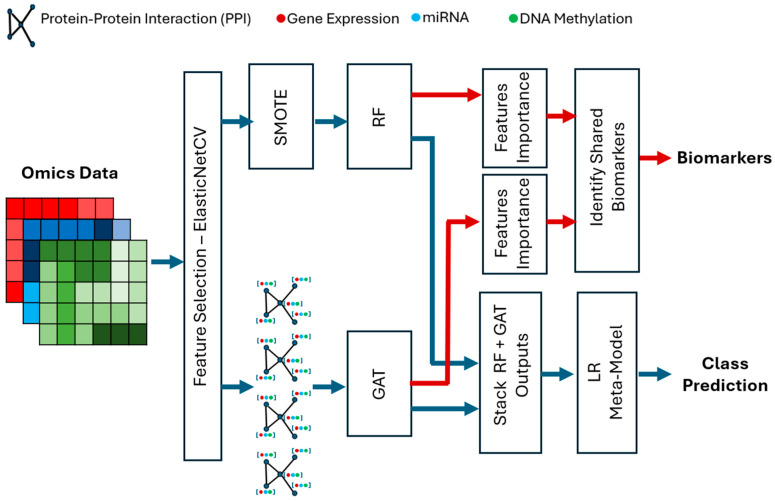
The hybrid multi-omics classification framework integrating ElasticNet feature selection, RF feature importance, and GAT-based graph learning with logistic regression stacking for biomarker discovery and class prediction.

The hybrid framework that is proposed is based on feature selection, graph learning, and stacking ensemble modeling to enhance multi-omics classification and biomarker discovery. First, ElasticNet is used on mRNA, miRNA, and DNA methylation data to narrow down the most informative and nonredundant features, which is essentially a dimensionality reduction step. The chosen features are subsequently subjected to two complementary models, an RF which scores the importance of the features used in the identification of biomarkers and a GAT which is based on a Protein–Protein Interaction (PPI) network that can learn the relational patterns between the genes. Logistic Regression stacking is used to combine prediction outputs of both models to increase accuracy of the classification and stability of the models. RF feature importance is used to identify the most important biomarkers, which are then selected based on topological relevance in the GAT and offer interpretable information regarding the biological process involved. Figure 1 shows the overall framework.

In this paper a rigorous experimental design was carried out where the multi-omics data (mRNA, miRNA, and DNA methylation) were combined to classify lower-grade glioma and ensure that data leakage would not occur. All preprocessing steps, including normalization, variance filtering, ElasticNet feature selection, and SMOTE balancing, were confined to the training folds within a repeated stratified 5-fold cross-validation scheme to ensure unbiased evaluation.

ElasticNet was used with a mix of L1 (Lasso) and L2 (Ridge) penalties with a mixing parameter 0.1 (l1ratio) and a grid of 50 logarithmically spaced regularization strengths of $10^{-5}\;to\;10^{0}$. Each training fold had a 5-fold cross-validation to determine the optimal penalty that minimizes the mean squared error to stabilize sparsity of the features and avoid overfitting. To alleviate the effects of the imbalance in the classes, we employed the Synthetic Minority Over-Sampling Technique (SMOTE) using k = 5 nearest neighbors, which is normally performed under the biomedical setting where the minority sample is small [26]. The degree of oversampling was controlled to ensure that there is a variety of classes but does not cause significantly close synthetic instances to be introduced. The choice of these parameters was empirically checked to balance the model robustness, biological understanding, and computational cost while minimizing potential selection bias.

### 2.4. Graph Construction

To model patients on a graph-based architecture, we combined multi-omics features and prior biological knowledge on protein–protein interaction (PPIs). The PPI priors were curated from the STRING v12.0 database https://string-db.org (accessed on 8 September 2025) using its RESTful API for Homo sapiens (taxonomy ID 9606). To have biological reliability, only the interactions with a confidence score of 0.4 (400/1000) or higher, which is medium-to-high confidence with either experimental or database evidence, were retrieved. This threshold is selected based on previous omics studies which showed that this threshold is an optimal choice [27,28]. A more conservative network would be obtained by setting a higher threshold (e.g., −0.7) and a more liberal network would be obtained by setting a lower threshold (<0.4). However, excessively low thresholds may introduce noisy or spurious edges. We used a batch of 100 genes at a time to stay within API limitations, and all the interactions returned were combined and de-duplicated based on unique gene-pairs to eliminate duplicate or symmetric edges. Only the edges that involve genes that are in the annotated multi-omics dataset were selected to eliminate redundant nodes that were not reflected in our transcriptomic, methylation, or miRNA data. These steps produce a biologically significant and non-redundant PPI network, which is the structural prior used by the GAT learning procedure. A patient was represented as a graph, with the genes forming the node and the functional association formed by the STRING database as the edges. The topology of the PPI network (edges) of all patients is the same, indicating the global protein interaction network provided by the STRING database. This is inspired by the work of [29], which used the same graph topology for all samples. In the case of individual patients, however, the node feature matrix is customized to the specific multi-omics profile (mRNA, miRNA, and DNA methylation) by mapping it to the corresponding set of genes. Missing omics modalities were filled with zeros to ensure uniform dimensionality across nodes. Through this design, graph-based architecture will be able to learn representations that not only reflect molecular alterations but their topological context within biological interaction networks.

### 2.5. Graph Attention Network (GAT)

The GAT is a neural network that is designed to carry out the task of representation learning on a graph by using an attention mechanism to emphasize the significance of neighboring nodes when passing the message [30,31]. We have a graph G=(V,E) where V is a set of nodes (i.e., genes) and E is the set of edges (i.e., interactions). Each node i has a feature vector (i.e., omics features) and can be given as $h_{i}\in R^{F}$, where F is the number of input features per node. The linear transformation of the node features can be given as

(1) $$h_{i}^{'}={Wh}_{i}\;\;\;$$

$W\in R^{F^{'}\times F}$ is a learnable weight matrix. $F^{'}$ represent the output features dimension. The attention coefficient between a node i and its neighbor j is computed as

(2) $$e_{ij}=LeakyReLU\left(a^{T}\left[h_{i}^{'}||h_{j}^{'}\right]\right)$$

where $a\in R^{2F^{'}}$ is a learnable attention vector and ∥ denotes vector concatenation. To compute the final attention weights, these raw coefficients are normalized among the neighbors of node *i* with the use of a softmax function as follows:

(3) $$\alpha_{ij}=\frac{exp(e_{ij})}{\sum_{k\in N_{i}}exp(e_{ik})}$$

where $N_{i}$ is the set of neighbors of node i. The node *i* final output representation is then calculated as a weighted sum of the features of its neighbors using the following equation:

(4) $$h_{i}^{\left(l+1\right)}=\sigma \left(\sum_{j\in N_{i}}\alpha_{ij}h_{j}^{'}\right)$$

where σ (.) represents a nonlinear activation function. To improve stability and expressiveness, multi-head attention is applied by repeating this process *K* times and either concatenating or averaging the results:

(5) $$h_{i}^{\left(l+1\right)}={\left|\right|}_{k=1}^{K}\sigma \left(\sum_{j\in N_{i}}\alpha_{ij}^{\left(k\right)}W^{\left(k\right)}h_{j}\right)$$

where K is the number of attention heads. In this manner GAT can dynamically learn which links are more powerful to each node and it allows it to learn context-dependent and biologically meaningful relationships in complex molecular networks.

In this paper the parameters of the GAT model (number of attention heads = 8, hidden channel size = 64, dropout = 0.5, learning rate = 0.001, weight decay = 1 × 10^−4^) were optimized using a grid search approach on the aggregated training folds across all subtypes of LGG. This ensures consistent model architecture across all classes’ labels, and this enables the model to avoid overfitting subtype-specific cohorts and enable the comparison of performance across classes

### 2.6. Random Forest (RF)

The RF algorithm is an ensemble learning algorithm that helps to combine the performances of several decision trees to achieve a better classification and reduce overfitting [32]. The tree of decision in the forest is trained using a bootstrap sample of the original dataset, and at every node, a random subset of features is selected to find the optimal split. This randomization will make this tree a diverse one and it will be more effective in generalization.

For training set $D={\left\{\left(x_{i},y_{i}\right)\right\}}_{i=1}^{N}$ where $x_{i}\in R^{F}$ is the feature vector, $y_{i}$ is the class label, and $R^{F}$ is F-dimensional real-valued vector space; the RF constructs T decision trees $\{h_{t}(x){\}}_{t=1}^{T}$.

To obtain a final prediction, a majority voting is conducted across all trees using the following equation:

(6) $$\hat{y}=mode\left({\left\{h_{t}\left(x\right)\right\}}_{t=1}^{T}\right),$$

RF is also fully applicable to high-dimensional biological data (e.g., multi-omics data) as it can learn nonlinear relationships, handle correlated and noisy data and offers scores of feature importance based on the average reduction in impurity across trees. These measures of importance assist in the discovery of major biomarkers that are related to disease subtypes. RF remains a strong baseline and supplementary model in deep learning methods, such as GAT.

### 2.7. Synthetic Minority Over-Sampling Technique (SMOTE)

SMOTE [33] is an augmentation technique of data used in counterbalancing the class imbalance by creating fake samples of the minority group. Instead of duplicating existing samples, SMOTE generates new points along the line sections that connect a minority sample to its K -nearest neighbors within the feature space.

With a minority sample $x_{i}$ and one of its neighbors $x_{j}$, a new synthetic example $x_{new}$ is obtained as

(7) $$x_{new}=x_{i}+\delta \;.\;\left(x_{j}-x_{i}\right),$$

where δ∼U(0,1) is a random number taken from a uniform distribution. The SMOTE approach enhances the variety of the minority samples, equalizes the proportion of the classes and enhances the stability and generalization of the classifiers, particularly in biomedical and multi-omics datasets, which sometimes comprise a number of cases for one of the classes.

### 2.8. ElasticNet

The regularized linear model (ElasticNet) [34] is a hybrid of the Lasso (L1) and Ridge (L2) penalties to perform feature selection and avoid overfitting, especially in high-dimensional data. With features X and target values y dataset, ElasticNet approximates the coefficients β by minimizing the objective:

(8) $$L\left(\beta \right)=\frac{1}{2n}||y-X\beta {\left|\right|}_{2}^{2}+\;\text{ʎ}\left[\alpha ||\beta {\left|\right|}_{1}+\frac{1-\alpha }{2}||\beta {\left|\right|}_{2}^{2}\right]$$

where ʎ modulates the total strength of regularization, and α ∈ [0, 1] balances the contributions of the $L_{1}$ (sparsity) and $L_{2}$ (shrinkage) penalties. The $L_{1}$ term promotes sparsity by enforcing some coefficients to zero, allowing the automatic detection of features, and the $L_{2}$ term stabilizes the estimates in cases where features are strongly correlated. In multi-omics datasets, ElasticNet is an effective method in picking informative genes or molecular features, which add the most value to predicting disease subtypes, and is a powerful preprocessing step prior to machine models [35,36].

### 2.9. Logistic Regression Meta-Model

The stacking ensemble of the proposed framework uses Logistic Regression (LR) to be the meta-learner, as it would integrate the prediction probabilities of the RF and GAT models. LR estimates the dependence between predictors of input (probabilities of the model) and the prediction of the output classes through the logistic (sigmoid) function. The likelihood of the sample in class y=1 is as follows [37]:

(9) $$p\left(y=1|x\right)=\frac{1}{1+e^{-(\beta_{0}+\beta_{1}x_{1}+\beta_{2}x_{2}+\cdots .\beta_{n}x_{n})}}$$

$x_{i}$ represent the input features (the outputs of the base models in this case), and $\beta_{i}$ are the learned coefficients. These coefficients are optimized to separate the classes by giving more effective weight to the contribution of each base learner for more accurate and more stable final predictions.

## 3. Results

**Figure 2:**
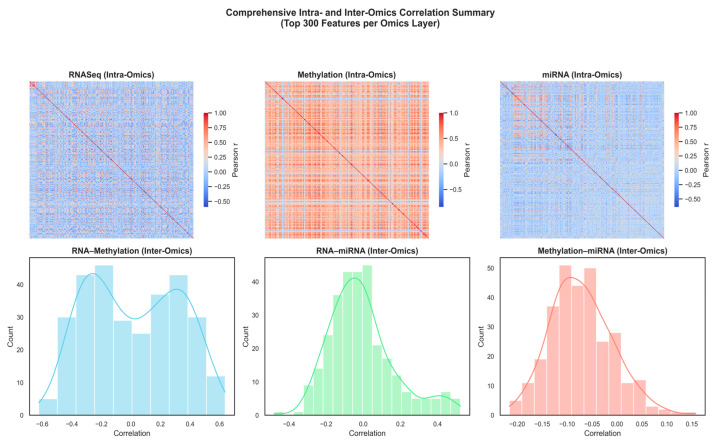
Comprehensive intra- and inter-omics correlation summary across RNA-Seq, DNA methylation, and miRNA layers (Top 300 features per layer).

We analyzed the correlations between the 300 features of each dataset that were most varied across the samples to investigate how the various omics layers complement each other. The features that characterize RNA-Seq, DNA methylation, and miRNA profiles were selected due to their high variability, implying greater biological activity and higher likelihood of differentiating between sample groups. Through the emphasis of these highly dynamic genes, CpGs, and miRNAs, the visualization reveals the structural and functional interaction between the three omics layers. As shown in Figure 2 (upper panels), the intra-omics correlation heatmap of RNA-Seq and DNA methylation demonstrates dense positive correlation, which indicates coordinated epigenetic regulation and coherent gene co-expression. The heatmap also shows that the internal correlation of the miRNA is weak, which reflects its heterogeneous expression behavior and the diversity of its broader regulation. The inter-omics correlation distributions (lower panels) reveal positive and negative correlations between omic layers, especially between RNA-seq and methylation, which reveal a close relationship between transcriptional activity and epigenetic regulation in the low-grade glioma biology. This illustrates that each omic layer can provide complementary regulatory data, thus supporting the value of integrating multi-omics data to describe the complex molecular heterogeneity underlying glioma subtypes.

### 3.1. Impact of Multi-Omics Integration on LGG Subtype Classification

Table 1 shows the impact of the combination of multiple layers of omics on the performance of LGG subtype classification. Of all single-omic analyses, DNA methylation (METH) was the most accurate (0.980 ± 0.015) and demonstrated the overwhelming importance of epigenetic regulation to differentiate between glioma subtypes. RNASeq (RNA) was also powerful on its own (0.972 ± 0.015) as it captures the effect of transcriptional activation and repression patterns between subtypes. Conversely, miRNA (MIR) had smaller, yet significant, accuracy (0.923 ± 0.029), indicating that post-transcriptional regulation has a more subtle/indirect signal of subtype distinction.

The combination of two omics layers gave a further improvement. The combination of the RNA and METH had one of the highest dual-omic accuracies (0.981 + 0.015), indicating the complimentary character of transcriptional and epigenetic data, namely, data reflecting the activity of genes and data reflecting the silencing of genes through methylation. Equally, METH + MIR (0.981 + 0.013) had a high degree of synergy, indicating that the miRNA patterns partly reflect the epigenetics regulation, whereas RNA + MIR (0.968 + 0.015) had less synergy, indicating that there is a degree of redundancy between expression and miRNA signals.

The triple-omics integration (RNA + METH + MIR) was the most successful in most of the metrics (accuracy = 0.984 ± 0.012; F1 = 0.985 ± 0.012; precision = 0.985 ± 0.013; recall = 0.986 ± 0.011), as it gives the fullest molecular coverage of the subtypes of glioma. All these results support the important conclusion that multi-omics integration can contribute a great deal to the stability and predictive ability of the model more than a single-omic method. These findings highlight that the DNA methylation is the key molecular predictor of subtype heterogeneity, whereas RNASeq and miRNA layers of heterogeneity offer additional data that enhances the generalization and biological understanding.

This is in line with earlier studies that have shown that DNA methylation dominates in the determination of molecular subtypes and the heterogeneity of tumors. Indicatively, Shi et al. (2020) demonstrated that methylation-based classification discriminates better subtypes and prognosis than transcriptomic layers in lung adenocarcinoma [38]. In a similar vein, Ma et al. (2024) have observed that immune microenvironment and phenotypic diversity in glioma are closely correlated with variations in DNA methylation patterns, which can be used to emphasize the core of molecular heterogeneity [39]. Also, integrative multi-omics analyses, including the one by Yang et al. (2024), have shown that DNA methylation is the main determinant of subtype structure, but transcriptomic and miRNA data can be used to improve biological interpretation and extrapolation of tumor samples [40]. This epigenetic plus transcriptomic interdependence has also been revealed in recent research on methylation-driven ceRNA networks (Li et al., 2025), where transcriptional layers are found to supplement core predictive ability of methylation in the discovery of tumor regulation networks [41].

**Figure 3:**
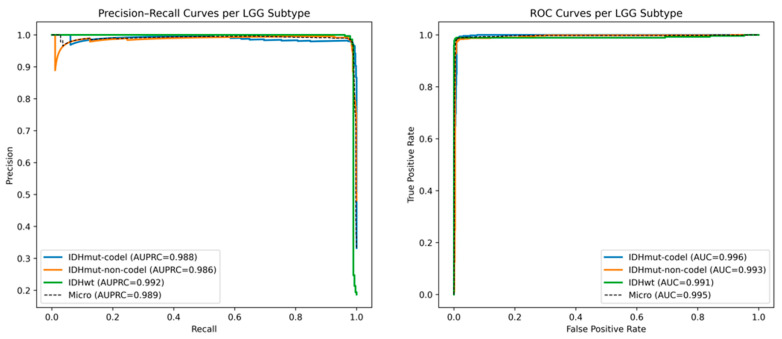
Precision–recall and ROC curves for LGG subtype classification using the RF-GAT hybrid model.

Figure 3 depicts the Precision Recall (PR) and Receiver Operating Characteristic (ROC) curves of the three major Lower-Grade Glioma (LGG) molecular subtypes, namely, IDHmut-codel, IDHmut-non-codel, and IDHwt, as predicted by the RF-GAT hybrid model. All the curves demonstrate very good classification performance on all the subtypes with AUPRC values more than 0.98 and AUC values higher than 0.99, which means a high sensitivity and good balance between precision and recall. IDHwt has the best AUPRC (0.992) and its AUC (0.991) representing strong discrimination between the mutant and wild-type classes. The almost ideal form of the curves and the low trade-off between precision and recall indicate the stability and the reliability of the model to capture the molecular signature that discriminate between LGG subtypes. This good performance shows that the combination of RF and GAT is an effective way to leverage both the topographical context obtained by local feature relevance and inter-gene topographical context obtained by the protein–protein interaction (PPI) network. The hybrid design minimizes the noises created by redundant features, as well as strengthens biologically significant relationships. In general, these findings prove that the RF and GAT hybrid model offers a discriminating and biologically interpretable model of LGG subtype classification, which can be used to support downstream activities, including prognostic modeling and biomarker discovery.

To determine the statistical significance of the improvements that were made by the proposed hybrid model, pairwise *t*-tests were conducted comparing the hybrid model with the baseline models (RF and GAT) with respect to the ROC-AUC and AUPRC metrics.

As shown in Table 2, In the case of ROC-AUC, the hybrid model was significantly better than the GAT baseline (t = 53.303, *p* < 0.001), whereas it was not better than RF (t = −1.827, *p* = 0.089). Similarly, in the case of AUPRC, the hybrid model was seen to perform greatly better than the GAT model (t = 88.567, *p* < 0.001) though the same is not true of the RF model (t = −1.740, *p* = 0.104). These results indicate that the hybrid model gives a statistically significant performance gain when compared to the GAT base model, and its performance is similar to the RF model. The insignificant variations with RF suggest that the hybrid model does not compromise the good predictive ability of RF, and it provides graph-level interpretation by feature learning via attention. This justifies the usefulness of hybrid approaches to multi-omics classification problems, where the combination of heterogeneous model views can stabilize performance across diverse data modalities.

### 3.2. Integrative Multi-Omics Clustering Based on Hybrid RF-GAT Selected Features

**Figure 4:**
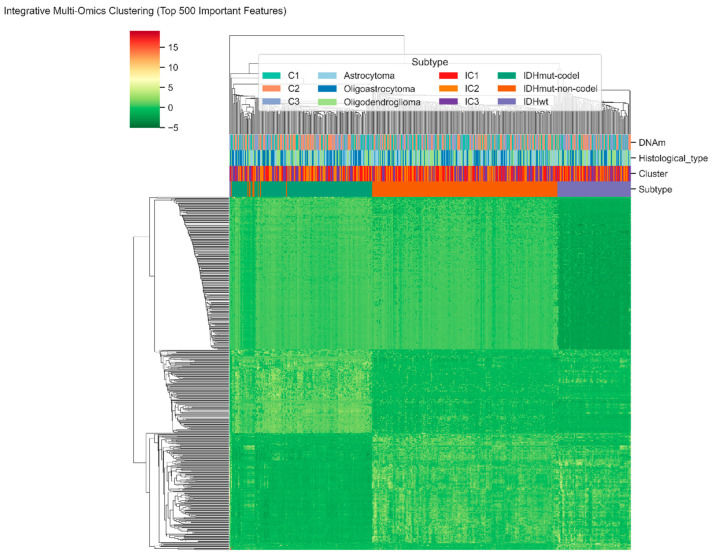
Integrative multi-omics clustering of lower-grade glioma samples based on the top-500 features identified by the hybrid RF-GAT model.

Figure 4 shows the integrative hierarchical clustering of the 500 most informative features of our hybrid model of RNA-seq. miRNA, and DNA-methylation data. All columns are patient samples of LGG and all rows are molecular features that are normalized by z-score transformation. The gradient in red-to-green coloring shows the level of relative expression or methylation and the bars above the heatmap are annotations of DNA-methylation clusters (C1–C3), histologic types, integrative clusters (IC1 3), and molecular subtypes based on IDH. The clear patterns of separation between these annotations indicate that the RF-GAT-generated multi-omics characteristics can capture the latent heterogeneity of lower-grade glioma at the molecular level. It is worth noting that the subgroups of IDHmut-codel, IDHmut-non-codel, and IDHwt are distinct groups, which proves the capacity of our hybrid model to identify biologically consistent and clinically meaningful trends based on transcriptomic and epigenetic levels.

The ElasticNet feature selection method has produced a stable collection of informative features between folds and, as such, it is stable and suitable for the high dimensional multi-omics data. In the repeated cross-validations, 189 to 401 features were selected on average per fold with an average of 43% RNA-Seq features, 54% DNA methylation features, and 3% miRNA features. This distribution indicates the greater discriminative role of transcriptomic and epigenetic data. The resulting reduced feature space was the input to the hybrid RF-GAT model, enabling effective learning of both discriminative and topological patterns with a biological interpretation.

We have also analyzed the correspondence between top-ranked features using the RF and the network topology using the GAT to understand the relationship between feature importance using both the RF and the network topology using the GAT-derived PPI graph. We found that some genes with large RF importance were also at the core of the graph structure, having large node degree and weights of the attention, indicating that not only do these genes play a role in discrimination of subtypes, but also form network hubs with important molecular interactions. The fact that feature-level importance and topological relevance are converged in hybrid model shows that the hybrid model combines the complementary points of view: the RF contains the discriminative power on the feature level, and the GAT contextualized these features in the biological network structure. This consistency adds to the biological plausibility of the discovered biomarkers and emphasizes the interpretability benefit of the proposed RF-GAT hybrid approach.

### 3.3. Identification and Biological Interpretation of Key Biomarkers Using the RF-GAT Hybrid Model

**Figure 5:**
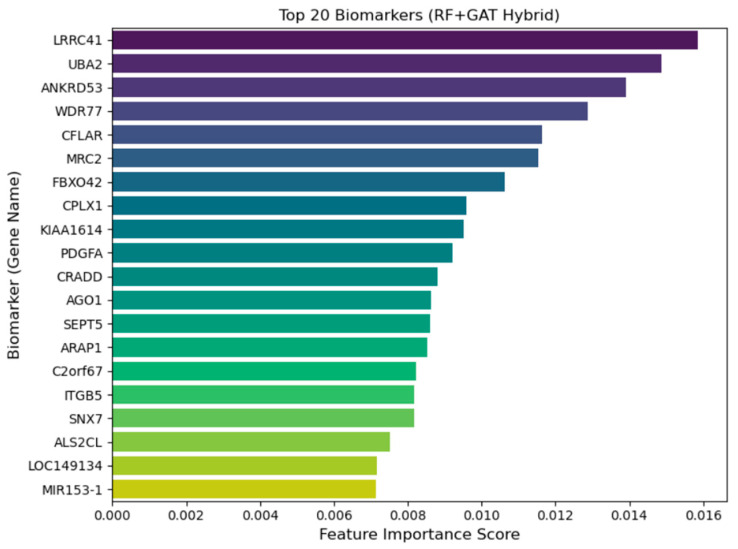
Top-20 candidate biomarkers contributing to LGG subtype discrimination identified by the RF+GAT hybrid framework.

Figure 5 shows the top 20 biomarkers and their feature importance obtained scores using the RF-GAT hybrid model. Biomarkers are the genes that most strongly contribute to the classification of lower-grade glioma (LGG) subtypes. The most important genes that show high feature important scores are LRRC41, UBA2, ANKRD53, and WDR77. The features important score obtained by these genes indicate their influence on the decision-making process of the model. Interestingly, some of these genes, e.g., LRRC41 and UBA2, have been identified previously as being related to tumor progression, cell cycle and ubiquitination pathways, and altered expression of these genes could be a cause of glioma pathogenesis. Ou et al. (2024) have shown that the UBA2 expression is associated with tumor progression, immune modulation, and radiosensitivity in glioma, and suggested it as a prognostic biomarker and a potential treatment target [42]. In addition, SUMOylation by UBA2 is also operative in glioma: UBA2 and its target RALY are increased in glioma cells and UBA2 knockdown inhibits vasculogenic mimicry and glioma cell proliferation [43]. Conversely, there have not yet been direct functional studies of the involvement of LRRC41 in glioma; LRRC41 protein staining of glioma tissue is not detected on the Human Protein Atlas. Nevertheless, LRRC41 has been involved in cancer pathways elsewhere (e.g., hepatocellular carcinoma), and one integrative multi-omics study has identified that LRRC41 is co-expressed with a block of genes that are negatively related to tumor phenotypes [44]. Therefore, although the role of UBA2 in glioma is corroborated by new empirical evidence, the role that LRRC41 plays in glioma is more hypothetical and should be further functionalized.

ANKRD53 is reported to have a role in regulating mitotic metaphase alignment, microtubule dynamics, and cytokinesis, and its malfunction may result in chromosome misalignment, spindle checkpoint activation, and cytokinesis failure [45]. Even though there are no direct signals of ANKRD53 involvement in glioma, its functions in mitotic fidelity position it as a candidate gene in tumor evolution. However, WDR77 has clear links to glioma since it is overexpressed in higher-grade and mesenchymal gliomas and is co-expressed with genes involved in cell cycle, metabolism, and immune regulation in glioma cohorts; therefore, it correlates with worse survival outcome [46].

## 4. Discussion

The hybrid score of the RF and GAT components indicates the complementary action of local feature selection and graph-based relational learning. RF can capture the statistical value of individual features, whereas GAT is able to model inter-gene relationships based on the attention mechanisms dictated by the protein–protein interaction (PPI) network. This shows that the hybrid approach improves the interpretability and biological relevance of features by determining biomarkers that are topologically central in the molecular interaction space.

It is interesting that both epigenetic (methylation) and transcriptional (RNA expression) biomarkers are among the top-ranked features, and that shows the integrative power of multi-omics analysis and how changes in methylation patterns (e.g., CFAR, MRC2, CPLX1, KIAA1614) are compensated by changes at the expression level to narrow down the discrimination of subtypes. Together, these findings emphasize a strong group of possible diagnostic and prognostic biomarkers of LGG that integrate network and molecular level insights.

Table 3 provides the statistical support of which omic layer (RNASeq or DNA methylation) best distinguishes between LGG subtypes of each of the top 10 biomarkers. The analysis indicates that four genes (LRRC41, UBA2, WDR77, and FBXO42) are most subtype specific at the transcriptomic (RNASeq) level, and six genes (ANKRD53, CFLAR, MRC2, CPLX1, KIAA1614, and PDGFA) are most altered by tailored epigenetic (methylation) changes. The *p*-values are extremely small ($6.86\;\times \;10^{-77}$ to $2.96\;\times \;10^{-280}$), which implies very strong statistical significance and shows that both omics layers discriminate against subtypes.

From a biological perspective, the fact that most of the biomarkers characterizing lower-grade glioma subtypes involve methylation underlines the central role of epigenetic regulation in the differentiation of the subtypes, especially those processes mediated by IDH mutation and 1p/19q codeletion, which have been shown to cause global reprogramming of methylation. Low *p*-values (as in the genes KIAA1614 and MRC2) indicate a significant form of silencing or activation of the genes by methylation that can lead to tumor behavior and prognosis. On the other hand, the transcriptional upregulation of genes identified using RNASeq-based methods including UBA2 and WDR77 are significantly increased in distinct subtypes, indicating that transcriptional reactivation or increased production of mRNA can be a driver of oncogenic activity in IDHmut tumors. This suggests that transcriptomic dysregulation and epigenetic dysregulation can play an important role in glioma heterogeneity, although mechanisms based on methylation seem to be slightly more influential in distinguishing between the subtypes.

**Figure 6:**
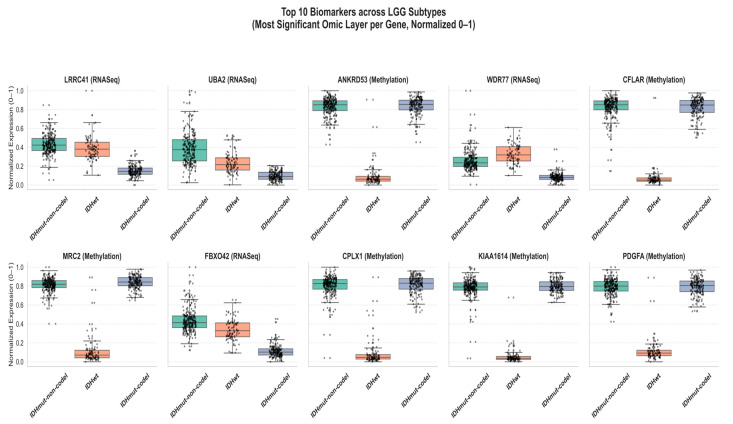
Differential expression of the top 10 biomarkers across LGG subtypes (normalized 0–1 per gene).

Figure 6 shows the patterns of expression of the top ten biomarkers found in the integrated multi-omics study of lower-grade glioma (LGG) on a normalized basis. The box plots depict the distribution of the expression of a biomarker among the three major subtypes of LGG, IDHmut-codel, IDHmut-non-codel, and IDH-wildtype (IDHwt) using the most informative layer of omic (RNASeq, DNA methylation, or miRNA) based on the lowest ANOVA *p*-value. All the values were minmax normalized to 0–1 to be compared across various omic modalities. The results indicate distinct patterns of subtype-specific gene expression and DNA methylation, with relatively limited molecular variability observed within the LGG subgroups. The biomarkers (RNASeq-derived) LRRC41, UBA2, and WDR77 are more expressed in IDHmut-non-codel compared to IDHwt, suggesting that such molecules can be part of the tumor development through transcriptional enhancing mechanisms. On the other hand, the biomarkers exhibiting stronger gain of IDHwt methylation enhancements (ANKRD53, CFLAR, MRC2, CPLX1, KIAA1614, and PDGFA) hint to the fact that more aggressive tumors experience epigenetic silencing. As a rule, IDHwt samples have the minimum normalized values, which indicate either repression or hypermethylation of genes and are associated with poor prognosis. Taken together, the figure shows that the combination of multi-omics data helps improve the capacity to differentiate LGG subtypes and illustrates the complementary role of transcriptomic and epigenetic data in defining the heterogeneity of glioma and identifying possible diagnostic and prognostic biomarkers. The absence of miRNA-derived biomarkers in the top-ranked features indicates that miRNA patterns of expression have a more indirect effect on subtype differentiation, and mRNA and DNA methylation may be the predominant molecular signatures that determine the biological diversification in LGG subtypes.

**Figure 7:**
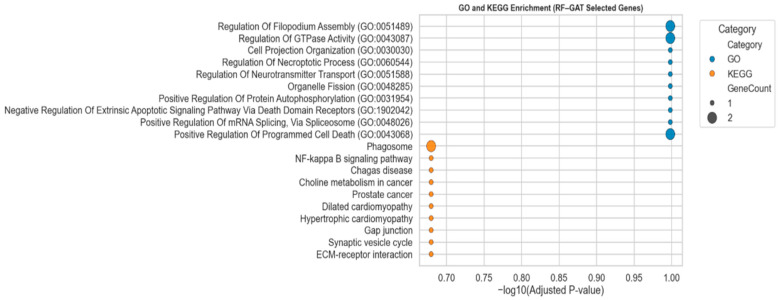
GO and KEGG enrichment of the top 20 biomarkers identified by the RF-GAT model. Bubble plot illustrates the top enriched GO biological processes (blue) and KEGG pathways (orange) based on −log_10_(adjusted *p*-value). The bubble size represents the number of genes contributing to each term. The 24 instances of the genes in the figure indicate overlapping genes that play a significant role in various terms.

To examine the biological relevance of the 20 biomarkers identified by the hybrid RF-GAT model, we conduct functional-enriched analysis, which is depicted in Figure 7. Even though the adjusted *p*-values were not below the standard significance levels, the Gene ontology (GO) and KEGG pathway enrichment findings showed coherent biological themes. GO biological processes were regulation of filopodium assembly, GTPase activity, neurotransmitter transport, mRNA splicing, and programmed cell death, which are closely related to cytoskeletal organization, intracellular transport, and apoptosis. Equally, NF-κB signaling, choline metabolism in cancer, and ECM–receptor interaction were identified in the KEGG pathway analysis, which has often been associated with tumor progression and microenvironmental regulation. These results imply that the best RF-GAT-learned biomarkers have functional meaning and reflect essential cellular processes in the development and aggressiveness of glioma.

As depicted in Figure 7, all the terms did not reach conventional statistical significance (adjusted *p* > 0.05), although the functional enrichment results revealed biologically coherent pathways associated with tumor progression and immune regulation. This can be attributed to the relatively small number of selected genes after multi-step feature selection and the conservative multiple-testing correction. Also, some of the keygens identified by the RF-GAT-based framework appear to be novel or less well-characterized and thus are not yet extensively annotated in GO or KEGG databases. This explains the non-significance of the adjusted *p*-value of these genes despite their potential biological relevance.

Though this study showed a high level of predictive performance based on TCGA-LGG cohort, the predictive performance of the results should be approached with caution. These analyses were performed on only one publicly available dataset, which might not fully represent the biological and technical differences that can be found between the different sequencing platforms, institutions, or patient groups. To overcome overfitting, we have used repeated five-fold cross-validation and ElasticNet regularization. External validation with independent datasets would also help to prove the strength of the proposed RF-GAT framework. As for future studies, we will focus on cross-cohort validation and the inclusion of multi-center data to evaluate the reproducibility and clinical transferability of the identified biomarkers.

## 5. Conclusions

This study introduces a biologically interpretable and computationally efficient framework for the molecular stratification of lower-grade glioma (LGG) subtypes using the joint analysis of multi-omics data with stacking ensemble of a protein–protein interaction (PPI)-aware GAT and Random Forest (RF) using logistic regression. The proposed model incorporating transcriptomic, epigenetic, and post-transcriptional profiles can effectively describe the interplay of complementary regulatory processes together with the heterogeneity of glioma. The findings show that the integration of multi-omics analysis significantly improves the predictive performance compared to unimodal, with an accuracy of 0.984 ± 0.012 at a high F1 and AUC results, indicating the synergy effect of transcriptional and epigenetic regulation. The attention-based interpretability analysis and scores of feature importance identified some important subtype-specific biomarkers, such as UBA2, WDR77, LRRC41, and ANKRD53 which have a high biological and clinical relevance and can be used as possible diagnostic or therapeutic targets. Both the modeling of molecular interactions and hierarchical dependencies, which the framework provides, allows us to understand the glioma pathophysiology on a deeper level and maintain a high level of classification stability.

Despite the promising results, several limitations should be acknowledged. The present study conducted analysis on one cohort (TCGA-LGG) and hence it needs to be validated on other datasets to establish its generalization. In addition, although transcriptomic, methylation, and miRNA layers were incorporated, other omics like proteomics and metabolomics were not used and may further contribute to the biological breadth of the model. The biomarkers were validated computationally, and their applicability as biomarkers needs to be confirmed experimentally or clinically. Lastly, the insufficient sample size might affect the stability of the model as well.

## Figures and Tables

**Table 1:** Summary of multi-omics combination performance.

Combination	Accuracy	Precision	Recall	F1	ROC-AUC	AUPRC
RNA	0.972 ± 0.015	0.975 ± 0.015	0.966 ± 0.022	0.970 ± 0.018	0.994 ± 0.006	0.989 ± 0.014
METH	0.980 ± 0.015	0.981 ± 0.015	0.982 ± 0.015	0.981 ± 0.015	0.995 ± 0.005	0.992 ± 0.008
MIR	0.923 ± 0.029	0.925 ± 0.025	0.913 ± 0.031	0.917 ± 0.028	0.986 ± 0.008	0.972 ± 0.014
RNA + METH	0.981 ± 0.015	0.983 ± 0.015	0.982 ± 0.015	0.982 ± 0.015	0.996 ± 0.006	0.992 ± 0.013
RNA + MIR	0.968 ± 0.015	0.971 ± 0.016	0.959 ± 0.023	0.964 ± 0.019	0.994 ± 0.006	0.988 ± 0.014
METH + MIR	0.981 ± 0.013	0.982 ± 0.014	0.984 ± 0.011	0.982 ± 0.013	0.995 ± 0.005	0.992 ± 0.008
RNA + METH + MIR	0.984 ± 0.012	0.985 ± 0.013	0.986 ± 0.011	0.985 ± 0.012	0.995 ± 0.005	0.989 ± 0.011

**Table 2:** Pairwise *t*-test results for performance comparison between hybrid and baseline models.

Metric	Comparison	*t*-Statistic	*p*-Value
ROC-AUC	Hybrid vs. RF	−1.827	0.089
ROC-AUC	Hybrid vs. GAT	53.303	0.000
AUPRC	Hybrid vs. RF	−1.740	0.104
AUPRC	Hybrid vs. GAT	88.567	0.000

**Table 3:** Most significant omic type per gene based on ANOVA *p*-values.

#	Gene	Best Omic	*p*-Value (Lowest)
1	LRRC41	RNASeq	$2.75\times 10^{-111}$
2	UBA2	RNASeq	$6.86\times 10^{-77}$
3	ANKRD53	Methylation	$6.63\times 10^{-256}$
4	WDR77	RNASeq	$6.39\times 10^{-85}$
5	CFLAR	Methylation	$3.44\times 10^{-247}$
6	MRC2	Methylation	$6.04\times 10^{-270}$
7	FBXO42	RNASeq	$7.88\times 10^{-115}$
8	CPLX1	Methylation	$5.39\times 10^{-233}$
9	KIAA1614	Methylation	$2.96\times 10^{-280}$
10	PDGFA	Methylation	$6.52\times 10^{-250}$

## Data Availability

The datasets generated and analyzed during the current study are available in GitHub repository at https://github.com/murtadaBashir/LGG (accessed on 11 October 2025).
